# Sleep disorder assessment in children and adolescents with neurodevelopmental disorders

**DOI:** 10.1016/j.jped.2025.101441

**Published:** 2025-09-26

**Authors:** Camila dos Santos El Halal, Magda Lahorgue Nunes

**Affiliations:** a Escola de Medicina da Pontifícia Universidade Católica do Rio Grande do Sul, Porto Alegre, RS, Brazil; b Instituto do Cérebro (InsCer), Porto Alegre, RS, Brazil

**Keywords:** Neurodevelopmental disorders, Sleep disorders, Sleep, Childhood, Adolescence

## Abstract

**Objective:**

To review the associations between various neurodevelopmental disorders and the most prevalent sleep disorders in children and adolescents, focusing on clinical characteristics and diagnostic approaches.

**Data sources:**

A literature review was conducted using the PubMed database, employing the search terms “neurodevelopmental disorders” and “sleep disorders,” including “insomnia,” “sleep-related breathing disorders,” “circadian rhythm sleep-wake disorders,” “sleep-related movement disorders,” “parasomnias,” and “central disorders of hypersomnolence.” Specific diagnostic terms related to neurodevelopmental and sleep disorders were also utilized. Additionally, the reference lists of selected manuscripts were manually reviewed to identify further relevant publications.

**Data synthesis:**

Sleep disorders are frequently associated with neurodevelopmental disorders such as autism spectrum disorder, attention-deficit/hyperactivity disorder, and intellectual disability, as well as genetic syndromes known to predispose individuals to sleep disturbances, including Down syndrome, Smith-Magenis syndrome, Prader-Willi syndrome, and mucopolysaccharidosis type II. These associations are mediated by anatomical, biological, and behavioral mechanisms. Diagnostic evaluation should be guided by clinical suspicion and typically involves a comprehensive clinical history, sleep diaries, standardized questionnaires, and, when indicated, additional diagnostic procedures.

**Conclusions:**

Sleep disorders are more prevalent in children and adolescents with neurodevelopmental disorders. A thorough understanding of the most commonly associated sleep disturbances and the implementation of targeted diagnostic strategies are essential for appropriate clinical management and for improving long-term outcomes in this population.

## Introduction

Neurodevelopmental disorders constitute a group of disorders that start early in an individual's life (in preschool age) and are characterized by developmental deviations that lead to impairments in social, personal, or academic functioning [1]. Examples include intellectual disability, autism spectrum disorder, and attention deficit hyperactivity disorder, as well as several genetic syndromes that may be characterized by one or more of these diagnoses, such as Down syndrome, Smith-Magenis syndrome, Prader-Willi syndrome, and Fragile X syndrome.

Comorbidities are common among patients with neurodevelopmental disorders, and sleep disorders are among the most prevalent ones. It is estimated that up to 86 % of children with neurodevelopmental disorders have sleep disorders, in contrast to neurotypical children, whose prevalence is around 25–30 % [2,3].

Sleep is involved in a series of physiological processes that affect neurodevelopment, such as brain plasticity, memory consolidation, emotional regulation, and cognitive functions [4]. The link between neurodevelopmental disorders and sleep disorders is multidirectional. Impairments in cognitive, social, communication, and physical functions present in neurodevelopmental disorders can alter sleep patterns and are also influenced by genetic, epigenetic, and environmental factors [3]. Poor sleep quality can also potentially compromise behavioral and neurodevelopmental aspects, further impacting these patients’ prognosis.

### Sleep disorders in children with neurodevelopmental disorders

Adequate assessment of sleep patterns and accurate characterization of the disorders are essential for the appropriate clinical management of patients with neurodevelopmental disorders. A sleep-related history should be part of the routine evaluation of patients with developmental disorders. This should be detailed and take into account various aspects that can influence sleep duration and quality, including prenatal and perinatal information, developmental milestones, characteristics of the sleep environment, and the individual's and family's sleep routines and habits (Table 1). A physical examination, assessing muscle hypotonia, changes in strength, craniofacial malformations, and obesity, is also essential.

**Table 1:** Suggested history for children and adolescents diagnosed with neurodevelopmental disorders and sleep complaints.

1. Gestational history, gestational age at birth, perinatal complications
2. Acquisition of development milestones
3. Sleep patterns from birth to the time of assessment
4. Presence of comorbidities
5. Screen exposure (hours per day and proximity to bedtime)
6. Waking routines: school schedule, extracurricular activities
7. Sleep routines: start time, presence of predictability, environmental conditions (brightness, absence of noise, adequate temperature), wake-up time on weekdays and weekends
8. Sleeping place
9. Conditions for falling asleep (presence of parents/caregivers, co-sleeping, sleep associations)
10. Sleep latency (time between lights out and falling asleep)
11. Nighttime sleeping location (individual room, shared room, shared bed)
12. Sleep characteristics (restless, peaceful)
13. Presence of snoring, mouth breathing, and respiratory pauses
14. Number and times of nocturnal awakenings
15. Characteristics of nocturnal awakenings
16. Easy or difficult waking up in the morning
17. Smoking or passive smoking, consumption of alcohol or other drugs
18. Chronic medications that may interfere with vigilance (antipsychotics, anti-seizure drugs, psychostimulants, antidepressants)
19. Frequency and time of physical activity
20. Symptoms of daytime sleepiness
21. Presence of daytime naps (number, time and duration)

The International Classification of Sleep Disorders divides sleep disorders into six categories, the relationships between which and some developmental disorders will be discussed below.

## Insomnia

Insomnia is defined as a persistent difficulty initiating or consolidating sleep, occurring despite adequate opportunity and circumstances, i.e., a suitable environment (appropriate lighting and temperature, absence of noise), leading to daytime impairments, such as behavioral changes and decreased academic performance [5].

In children diagnosed with autism spectrum disorder (ASD), insomnia is the most commonly reported sleep disorder, resulting in increased sleep latency (the time between lying down and falling asleep) and frequent nocturnal awakenings [6]. A British longitudinal study that followed participants from 2 years and 6 months to 11 years of age found that children and adolescents diagnosed with ASD had, on average, a 43-min shorter nighttime sleep duration compared to children with typical development [6]. This reduction was primarily due to later bedtimes and earlier awakenings, as well as multiple (at least 3) nocturnal awakenings. In another study, shorter continuous sleep time was associated with greater irritability and more stereotypies in this group of children [7]. Comorbidities frequently present in ASD, such as intellectual disability, deficits in communication skills, sensory processing disorder, ritualistic behaviors, and reduced responses to social cues, can contribute to the onset of insomnia [8].

It is estimated that sleep disorders are present in up to 55 % of children diagnosed with Attention Deficit Hyperactivity Disorder (ADHD) [9]. A meta-analysis using subjective and objective sleep data found that children with ADHD have greater resistance to falling asleep and difficulty initiating sleep, a greater number of nocturnal awakenings, greater difficulty awakening the following day, more symptoms of daytime sleepiness, and lower sleep efficiency assessed by polysomnography [10]. Sleep disorders can lead to exacerbation of ADHD symptoms, as can pharmacological treatment of ADHD symptoms, which first line of treatment includes psychostimulants that can lead to difficulty falling asleep, early awakenings, and restless sleep [10].

The presence of intellectual disability is also a risk factor for the development of insomnia. A study of children and adolescents (aged 5 to 18) diagnosed with intellectual disability found that the presence of comorbidities, especially recurrent pain, constipation, frequent coughing, or recurrent epileptic seizures, were more predictive of sleep disturbances than variables related to the individual's overall functioning (autonomy for communication, mobility, and self-care), highlighting the importance of actively investigating systemic symptoms in this group of patients [11].

The diagnosis of insomnia is clinical, requiring the presence of symptoms of difficulty inducing or maintaining sleep associated with daytime impairments for at least three days per week for at least three months. A guided history is therefore essential for a correct diagnosis and to rule out symptoms that suggest other sleep disorders, such as snoring and restless sleep. Sleep diaries, which consist of records of sleep data (time of falling asleep, number and circumstances related to nocturnal awakenings, time of awakening) as well as daytime activities, are typically completed for periods of at least two weeks. They allow the delineation of sleep patterns and aid in the diagnosis and development of a behavioral treatment plan [3].

Questionnaires can also be useful for assessing sleep patterns, although most are not specific to the diagnosis of insomnia. Among the options translated and validated for the Brazilian population is the BISQ (Brief Infant Sleep Questionnaire), which can be administered to children up to 3 years of age and takes into account patterns of sleep organization, duration of nocturnal and daytime sleep, associations for sleep onset, and parental perception of the child's sleep quality [12,13]. Between 3 and 18 years of age, a useful tool is the Children's Sleep Disturbance Scale, which assesses the presence of symptoms of insomnia and other sleep disorders, allowing diagnostic guidance and, when necessary, the investigation [14]. Among adolescents aged 12 to 18 years, the Epworth Daytime Sleepiness Scale for Children and Adolescents (ESS-CHAD) assesses the risk of falling asleep and, consequently, the degree of daytime sleepiness, helping to measure symptom severity [15].

Actigraphy can also be a useful tool for patients with insomnia, as it allows an objective assessment of the sleep-wake rhythm over prolonged periods. This wristwatch-shaped device, equipped with an accelerometer, records and stores movement data, generating information on wakefulness and, in moments of absence or reduced movement, on the individual's sleep. Its sensitivity is greater when used in conjunction with a sleep diary, allowing the distinction between periods of inactivity during wakefulness and inactivity during sleep [16].

## Sleep-related breathing disorders

Breathing disorders during sleep can occur due to obstructive apneas (obstructive sleep apnea/hypopnea syndrome – OSAS), central apneas, sleep-related hypoventilation disorders, or sleep-related hypoxemia [17].

OSAS is the most prevalent sleep-related breathing disorder, affecting 1 to 5 % of children. It is characterized by upper airway dysfunction, leading to partial or complete airway obstruction during sleep and a consequent reduction in oxygen saturation or sleep fragmentation [18]. Clinically, it may present through snoring and/or respiratory pauses during sleep, night sweats, or mouth breathing. Daytime symptoms of hypersomnia or behavioral (irritability, inattention, and hyperactivity) and cognitive (learning deficits) changes may also occur [19]. Genetic syndromes that present with hypotonia and craniofacial abnormalities favor the development of obstructive disorders. Trisomy 21 (Down syndrome – T21) is the most common hereditary chromosomal disorder, present in 1 in 700 live births. It presents with several potential comorbidities, of which OSAS is one of the most prevalent, estimated to affect between 50 and 100 % of individuals in childhood and close to 100 % of individuals in adulthood [20]. OSAS symptoms should be actively sought from the first years of life in children diagnosed with Trisomy 21. A randomized study including 40 children with T21 who underwent OSAS screening every six months between the ages of 6 and 36 months demonstrated that those diagnosed with OSAS who underwent early treatment had better developmental scores at 36 months [21].

The association between OSAS and ADHD has also been demonstrated in the literature. A meta-analysis conducted by Cortese et al. found a two-fold increased risk of sleep-disordered breathing among children with ADHD, although this association occurred in the analysis of subjective data [10]. In another meta-analysis, conducted by Sedky et al. and using only polysomnographic data, a moderate association was found between ADHD and OSAS, an association not mediated by body mass index [22]. In this same study, there was a reduction in ADHD symptoms after adenotonsillectomy.

Other genetic syndromes associated with craniofacial malformations (such as mucopolysaccharidosis type II) and obesity (Prader-Willi) are also frequently associated with sleep-disordered breathing, at a frequency of 77 % and 43 %, respectively [23].

The diagnosis of sleep-disordered breathing requires polysomnographic evaluation. Polysomnography uses several sensors, including airflow, snoring, and body position sensors, as well as strips for assessing respiratory effort and paradoxical breathing, oxygen saturation monitoring, and electroencephalographic electrodes for sleep staging. The diagnosis of OSAS in children requires an obstructive or mixed apnea or hypopnea index of at least 1 per hour of sleep. Another polysomnographic parameter is the presence of hypercapnia (PaCO_2_ > 50 mm Hg) for at least 25 % of total sleep time, in association with at least one of the following: snoring, nasal pressure wave flattening, or paradoxical thoracoabdominal movements [18].

## Circadian rhythm sleep and wake disorders

It is characterized by a misalignment between the endogenous circadian system and the external environment.[5]

Smith-Magenis syndrome (SMS), which includes intellectual disability, developmental delay, craniofacial abnormalities, aggression, and self-injurious behavior, almost always presents with circadian rhythm disorders, in addition to other sleep disorders. These patients have fragmented sleep, shortened sleep cycles, multiple nocturnal awakenings, and sleep-disordered breathing [3]. SMS results from a heterozygous deletion in the retinoic acid-inducible 1 (RAI1) gene, which is involved in the expression of CLOCK genes, which regulate sleep. Consequently, it is estimated that there is an alteration in the pattern of melatonin release, with peaks occurring during the day.

A meta-analysis comprising one hundred and three studies, fifteen of which addressed circadian rhythm disorders, found that such disorders are more frequent among individuals with ASD [8]. They also suggest that changes in the expression of CLOCK genes among these individuals may favor circadian dysregulation. Furthermore, there is evidence that irregularities in serum melatonin levels (delayed or reduced melatonin peak amplitude and alterations in the expression of melatonin-related genes) may also contribute to this dysregulation among individuals with ASD and may also be inversely related to the severity of autism symptoms [24].

Most of the evidence associating neurodevelopmental disorders with circadian rhythm alterations comes from studies of individuals with ADHD [25]. Such individuals have a tendency toward an evening chronotype, and in extreme cases, a diagnosis of delayed sleep phase syndrome may be possible [4]. A study including 34 children and adolescents diagnosed with ADHD and 43 controls found that melatonin levels fluctuated significantly between the groups between 10 and 12 years of age, with a reduction in its nocturnal signaling [26]. Another study evaluated the association between an evening profile and sleep disorders among adolescents (between 13 and 17 years old) diagnosed with ADHD [27]. The authors found that an evening pattern was associated with difficulties initiating and maintaining sleep, as well as with excessive daytime sleepiness. Also evaluating adolescents aged 16 to 19, a population-based study including 9,338 individuals, of whom 306 (3.3 %) met criteria for delayed sleep phase syndrome, found a 1.7-fold increased risk of ADHD symptoms in this subgroup [28].

Actigraphic monitoring is important for the diagnosis of circadian rhythm disorders, providing information on sleep and wake patterns over prolonged periods (ideally, two weeks). The American Academy of Sleep Medicine points out that the benefits of using actigraphy are its non-invasive nature, the possibility of prolonged monitoring of sleep parameters (which can be difficult for caregivers of children who rely on sleep diaries alone), its usefulness as a complement to self-assessment, and the increased accuracy that actigraphic data provide for both diagnosis and decision-making and monitoring of therapeutic response [29].

## Sleep-related movement disorders

The International Classification of Sleep Disorders describes this group of disorders as characterized by the presence of relatively simple, usually stereotyped, movements that interfere with sleep [5]. This group of disorders includes restless legs syndrome, periodic limb movements, sleep-related rhythmic movements, and bruxism, in addition to the recently described restless sleep disorder [5,30].

Restless legs syndrome (RLS) is a sensorimotor disorder characterized by an irresistible urge to move one's limbs, often accompanied by discomfort that worsens with rest and is relieved by movement. In these conditions, there is often concomitant insomnia secondary to limb discomfort and the need to move them. RLS is frequently found in individuals with ADHD, with a frequency of up to 44 % [31]. The association between ADHD and RLS seems to be bidirectional, with at least three hypotheses to explain their concomitant occurrence. The first considers that insomnia related to the need to move the limbs leads to sleep deprivation, with consequent symptoms of inattention, impulsivity, and hyperactivity during the day. The second hypothesis considers that daytime RLS symptoms can lead to ADHD symptoms, and the need to move the limbs may also be present during the day, leading to restlessness and difficulty remaining seated in class, as well as impaired attention. A third hypothesis considers that both ADHD and RLS may be clinical manifestations of a common dopaminergic deficit [32].

## Parasomnias

These are defined as physical events or experiences that occur during the transition from wakefulness to sleep, during sleep, or during the sleep-wake transition. Parasomnias that occur during non-REM sleep include sleepwalking, confusional arousals, and night terrors [5]. In childhood, the most prevalent REM sleep parasomnia is nightmare disorder. The literature describes that individuals with neurodevelopmental disorders have a higher risk of non-REM sleep parasomnias, possibly associated with an instability of this sleep pattern secondary to neurological disorders and neurotransmitter pathway dysregulation [4].

A study using polysomnographic sleep assessment found that, among 23 patients diagnosed with ASD, 14 had parasomnias on objective sleep assessment, 13 of whom had non-REM sleep parasomnias (sleep terrors or confusional arousals) [33].

Another study involving 2,284 students between 18 and 20 years of age found a significant association between sleep terrors and inattention symptoms among individuals diagnosed with ADHD (OR 2.4 – 95 % CI 1.3-4.5), as well as between sleep terrors and hyperactivity (OR 2.4 – 95 % CI 1.5-4.1) when compared to peers without the disorder [34].

Diagnosis is essentially based on clinical data. In situations where clinical description is not sufficient to determine the diagnosis, home videos made by parents may be helpful. Polysomnographic investigation should be limited to cases where a differential diagnosis with epilepsy is necessary, as well as when other comorbid sleep disorders are suspected [35].

## Hypersomnolence of central origin

The DSM-5 defines hypersomnolence as the presence of excessive daytime sleepiness despite a minimum of 7 h of nighttime sleep. This symptom must be associated with recurrent periods of irresistible sleep during the day, or an episode of sleep lasting more than 9 h, but not restful and/or accompanied by difficulty being fully awake after an abrupt awakening [1]. These include diagnoses such as narcolepsy, idiopathic hypersomnia, Kleine-Levin syndrome, and hypersomnias secondary to medical conditions, psychiatric disorders, or substance use.

The prevalence of narcolepsy among children and adolescents is unknown, but it affects between 25 and 50 adults per 100,000 individuals [36]. It is characterized by excessive daytime sleepiness accompanied by recurrent naps or irresistible episodes of daytime sleep. Type 1 narcolepsy is accompanied by cataplexy, characterized by brief episodes of bilateral muscle atonia without loss of consciousness, usually triggered by emotions, and by reduced cerebrospinal fluid (CSF) hypocretin concentrations. In type 2 narcolepsy, cataplexy is absent and hypocretin levels are normal. Diagnosis includes completion of a sleep diary associated with the use of actigraphy, polysomnography, and a multiple diurnal latency test. This involves daytime polysomnographic assessments of 20 min every 2 h, 4 to 5 times a day, starting approximately 2 h after awakening from a night of polysomnographic monitoring with at least 6 h of nocturnal sleep. A positive test demonstrates sleep latency of up to 8 min and more than 2 episodes of REM sleep (SOREMP). Lumbar puncture to assess cerebrospinal fluid (CSF) hypocretin levels and serum HLA-DQB1*0602 allele testing are also part of the routine investigation [36].

A systematic review of the literature, including ten studies and 839 patients diagnosed with narcolepsy, found an aggregate prevalence of 25 % (95 % CI 14-38 %) of ADHD in narcoleptic individuals, particularly among those with narcolepsy type 2. There is an overlap in symptoms between ADHD and narcolepsy, which makes their differentiation difficult. Symptoms of inattention, hyperactivity, and impulsivity can occur in narcolepsy, and excessive daytime sleepiness can mimic or exacerbate symptoms of ADHD. The mechanisms that associate one diagnosis to another may be genetic, biological, or environmental in origin [37].

Prader-Willi syndrome, which affects 1 in 15,000 to 30,000 live births and is characterized by hypothalamic-pituitary dysfunction, presents with feeding difficulties and severe hypotonia in the neonatal period, followed by a period of hyperphagia and excessive weight gain in childhood, often leading to obesity. It also presents with intellectual disability and behavioral problems. In this group of patients, excessive daytime sleepiness is a prevalent and disabling symptom. Although OSAS is common among these patients, there are also high rates of central apnea, and the main hypothesis is that hypersomnia in this group of individuals is primarily due to hypothalamic dysfunction, leading to reduced arousal mechanisms and abnormal ventilation during sleep [38].

Kleine-Levin syndrome (KLS) is a rare neurological disease of unknown origin characterized by episodes of hypersomnia associated with cognitive and behavioral disorders. It affects approximately 1–2 million individuals, predominantly males (68–78 % of cases) and adolescents (81 % of cases; mean age of onset is around 15 years). It is characterized by episodes of hypersomnia (with sleep periods lasting 15–21 h per day), cognitive impairment (apathy, confusion, sluggishness, and amnesia), and a feeling of derealization (a sleep-like state with altered perception). Other associated symptoms may include hyperphagia (66 %), hypersexuality (53 %, mainly men), anxiety, compulsive or mood changes, and depression (53 %, predominantly women). Between episodes, sleep, wakefulness, mood, and eating habits are similar to normal; however, patients with KLS have a higher body mass index. The disease course is 8 to 14 years [39].

## Conclusion

Sleep disorders are more prevalent among individuals with neurodevelopmental disorders than in the general population. However, their diagnosis can be difficult due to communication difficulties, intellectual impairment, or other comorbidities present in these individuals.

Understanding the most common disorders, conducting an adequate and systematic history in this group of patients, and conducting a targeted investigation are essential for adequately characterizing the disorders, their management, and potentially improving the quality of life of these patients.

## Conflicts of interest

The authors declare no conflicts of interest.
